# Affordable Phenotyping at the Edge for High-Throughput Detection of Hypersensitive Reaction Involving Cotyledon Loss

**DOI:** 10.34133/plantphenomics.0204

**Published:** 2024-07-17

**Authors:** Mathis Cordier, Pejman Rasti, Cindy Torres, David Rousseau

**Affiliations:** ^1^ Laboratoire Angevin de Recherche en Ingénierie des Systèmes (LARIS), UMR INRAe IRHS, Université d’Angers, Angers, 49000, France.; ^2^ R&D Artificial Vision and Automation, Vilmorin-Mikado, La Ménitré, 49250, France.; ^3^ Centre d’Études et de Recherche pour l’Aide à la Décision (CERADE), ESAIP, Saint-Barthélemy-d’Anjou, 49124, France.

## Abstract

The use of low-cost depth imaging sensors is investigated to automate plant pathology tests. Spatial evolution is explored to discriminate plant resistance through the hypersensitive reaction involving cotyledon loss. A high temporal frame rate and a protocol operating with batches of plants enable to compensate for the low spatial resolution of depth cameras. Despite the high density of plants, a spatial drop of the depth is observed when the cotyledon loss occurs. We introduce a small and simple spatiotemporal feature space which is shown to carry enough information to automate the discrimination between batches of resistant (loss of cotyledons) and susceptible plants (no loss of cotyledons) with 97% accuracy and with a timing 30 times faster than for human annotation. The robustness of the method—in terms of density of plants in the batch and possible internal batch desynchronization—is assessed successfully with hundreds of varieties of Pepper in various environments. A study on the generalizability of the method suggests that it can be extended to other pathosystems and also to segregating plants, i.e., intermediate state with batches composed of resistant and susceptible plants. The imaging system developed, combined with the feature extraction method and classification model, provides a full pipeline with unequaled throughput and cost efficiency by comparison with the state-of-the-art one. This system can be deployed as a decision-support tool but is also compatible with a standalone technology where computation is done at the edge in real time.

## Introduction

Selective plant breeding, or plant selection or plant improvement, is the process by which humans modify and create new plant species. It started 10,000 years ago with the domestication of wild plants and is now used to face the consequences of climate change [1]. The selected characteristics can include the resistance to biotic and abiotic stresses expected in the years to come, the capability to germinate earlier in the year to harvest before the chaotic ends of summer, and the nutritive and environmental values. For a few decades already, this process of selection has tried to anticipate the consequences of climate change and reduce the expected yield drop in agriculture [2]. However, producing a new variety is a long process that can take up to 10 years [3]. An approach to speed up this process, called breeding and prebreeding, consists of producing fast tests on a large number of candidates to select the most promising varieties. Such experiments can be done in controlled conditions to mimic climate scenarios or stress conditions [4]. In this context of breeding and prebreeding, imaging and machine learning can lead to speed up these tests with their power of parallelization. This article contributes in this trend. We focus on the phenotyping system, Phenogrid, that we have developed for plant phenotyping in prebreeding [5–7]. Phenogrid is composed of a network of mini-computers coupled with low-cost cameras gazing plants from top view in growth chambers or greenhouses. The images captured by this system can then be preprocessed to extract time-lapse images of the development of the individual plants. Such analysis is for instance accessible via the software Growth Data [8]. Phenogrid was initiated for the monitoring of the early stages of seedling development [5–7] under normal growth conditions or abiotic stresses. At these early stages, seedlings do not overlap and an individual monitoring of the plant is possible. In this article, we consider phenotyping situations where plants are grown under a biotic stress and at developmental stages where they overlap, extending the value of Phenogrid to these conditions.

One of the main criteria in plant breeding is resistance to pathogens that cause major yield losses and the substantial use of pesticides. Plant pathology is inherent to variety selection, in order to introduce or conserve resistance genes in the varieties produced. A wide diversity of susceptibility and resistance reactions exist. In this article, we focus on hypersensitive reactions [9,10]. These local defense mechanisms consist in destroying the cells at the site of infection in order to limit spread to the rest of the plant. Each pathosystem is specific, with different reaction types and durations. One of the most commonly observed symptoms is the appearance of necroses on leaves. Necroses are the result of premature cell death due to infection of the host plant by a pathogen [11]. Visually, they may have different shapes, colors, or textures, enabling the pathogen to be identified by pathologists. This type of reaction is widely targeted by research teams in their quest to automate the detection of these necroses, achievable via conventional imaging techniques. Much work is being done in computer vision to be able to detect these necroses via segmentation models [12–14] or to directly quantify severity [15–17].

For some pathosystems, the entire organ affected by pathogen infection can be destroyed in order to block propagation to the rest of the plant. This reaction mainly appears at the cotyledon level with cotyledon weakening and death. Some viruses can cause such reaction with *Tomato Spotted Wilt Virus* (TSWV) [18,19], *Pepper Mild Mottle Virus* (PMMoV) [20,21], or *Tobacco Mosaic Virus* [20,21], and fungi with *Fusarium* [22–24], *Verticillium* [25], or *Fulvia fulva* [26]. We propose to address this reaction by investigating the ability of temporal signals with the depth imaging modality to detect the loss of these dying cotyledons.

Depth time sequences can be considered as videos, with a relatively high sample rate compared to the speed of plant development. In the field of video classification, there are several deep learning models that have been proposed in recent years, each with its own strengths and weaknesses. One such type of model is the CNN-LSTM architecture [27–29], which combines the strengths of both convolutional neural network (CNN) [30–32], and long short-term memory (LSTM) [33–36] networks to respectively encode spatial and temporal dimensions. Similarly, the CNN-GRU [37] model also combines the strengths of both CNN and gated recurrent units (GRU) [38,39], to perform video classification tasks. Another type of model exists, using convolutions also for temporal dimension, called 3D-CNN [40,41] with 2 spatial dimensions and 1 temporal dimension. While these models can be highly accurate, they are also extremely heavy and computationally intensive. The choice of depth of the model layers and the model architecture itself affects the number of parameters to be optimized and the training time. Whatever the choice of these parameters, the use of such models requires a large amount of data for the training and implies a high energy consumption. The model we propose in this article is very lightweight compared to deep learning models. Sparse spatiotemporal encoding strategy is more computationally efficient while still achieving high accuracy in such video classification tasks. This lightweight model can be implemented at the edge, directly on mini-computers embedded in imaging systems. The performance of such electronic systems is too limited to support huge deep learning video classification models. Tiny versions of these video classification models are provided for inference on edge devices [42], but they come with impaired performance. This justifies the development of the proposed method for low-cost imaging systems, as the deployment of deep learning methods at the edge would require the use of a graphics processing unit or more expensive electronic components in general.

In varietal testing, a high density of plants is studied simultaneously to guarantee high throughput. This goes with strong overlapping between plants and leaves. Current imaging techniques do not yet provide an efficient individualization of plants and leaves in such very dense canopies corresponding to the throughput we will experience in this article [43–45]. By contrast with classical approaches [6,46–49], we propose not to attempt to individualize touching plants and rather to use the experimental protocol of grouping plants of the same variety in batches to carry out our diagnoses directly at batch scale.

The article is structured as follows. We first introduce the materials with a description of the imaging system used, the experimental protocol, and the datasets obtained. Secondly, the methods for qualitative signal analysis, extraction of characteristic spatiotemporal features and classification of batches of resistant and susceptible plants are detailed. Then, the results on feature extraction, the classification model and the robustness of the methods are described. Finally, the proposed method will be discussed on the basis of its performance and its ability to be embedded at the edge.

## Materials and Methods

### Experimental protocol

**Fig. 1.**
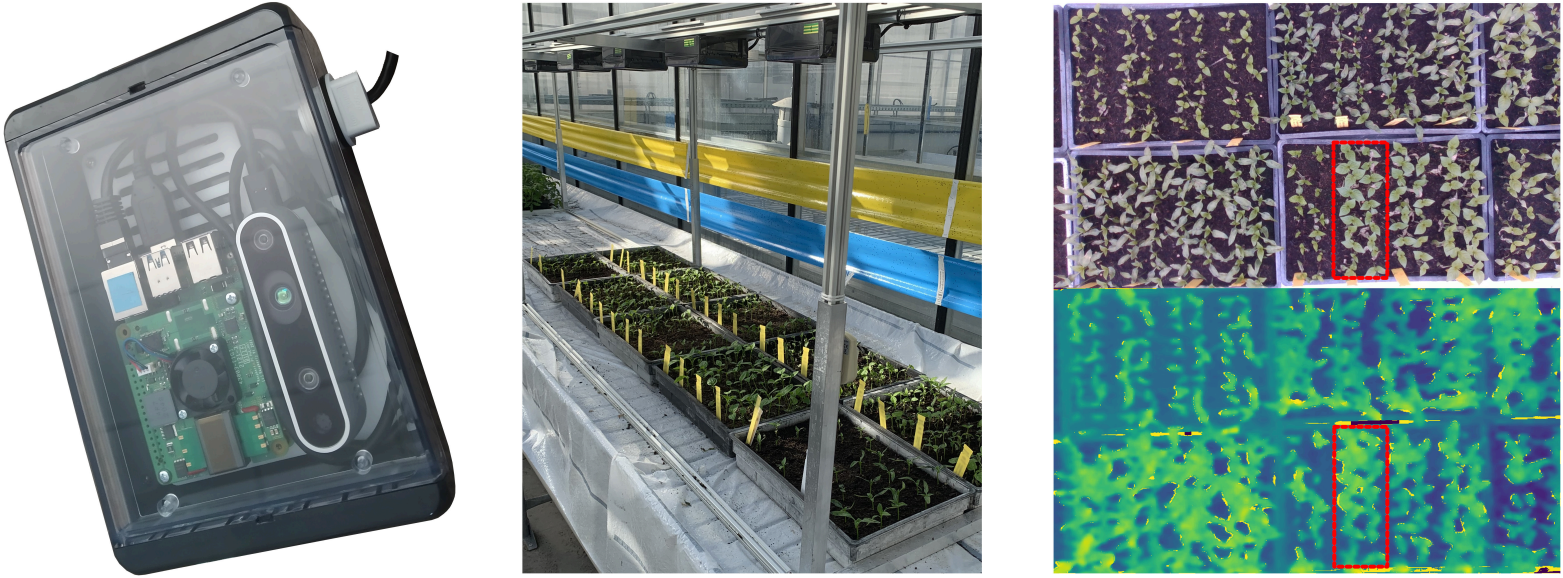
On the left, hermetic case composed of a mini-computer and a RGB-Depth camera. In the middle, view of system monitoring plant growth during a pathology test. On the right, example of RGB-Depth image in which plants of the same variety are grouped together in 2 rows of 10 plants within plant batches, an example of which is framed in red on both RGB and Depth image.

Resistance testing of pepper varieties is carried out in confined greenhouse rooms. The growth environment is therefore controlled so as to be initially pathogen-free and then in the sole presence of the pathogen under test. Plants are inoculated at the growth stage corresponding to the appearance of the first leaves. The 2 cotyledons of each plant are rubbed with a solution containing the pathogen so that it penetrates the lesions created. Plants of the same variety repetition are grouped together in a plant batch with a high seeding density with about 10 plants/dm ^2^. As visible in Fig. 1, plants from different batches are therefore spaced as far apart as possible to minimize overlap between the plant batches. The conjoint timing of sowing and inoculation for all plants guarantees homogeneous reactions. In particular, a maximum difference of 1 min is observed between plants of the same batch for their sowing and inoculation.

Pathologists diagnose the plants after a test period postinoculation. The duration of the test is highly dependent on pathosystem and growing conditions, such as light, temperature [50], and humidity, since these have an impact on the development of both the host plant and the pathogen. The duration corresponds to the time required for resistant plants to set up the physiological processes in hypersensitive reaction [9,10].

In our context, resistant plants destroy infected leaves, i.e., cotyledons [19]. Susceptible plants show no reaction over the observed period. Their growth is therefore normal over the test period. If this period were extended, the pathogen would contaminate the whole susceptible plants until their death. Pathologists base their diagnosis on the loss of cotyledons of each plant at the end of the test period. Information is then fed back to each batch of plants and finally to each variety to obtain a final diagnosis of susceptible and resistant varieties.

We propose to automate this experimental protocol. The absence of a robust method for individualizing plants in these high-density scenes with affordable imaging systems makes it necessary to monitor plants at the scale of batches of plants of the same variety. We investigate how to characterize the hypersensitive reaction involving cotyledon loss at the batch scale. This parallelization enables high throughput without the need for individual plant scoring prior to overall batch scoring.

### Imaging system

Automating plant monitoring necessitates an imaging system capable of acquiring images of plants over time. Such system was developed with a low-cost multimodal sensor, Intel RealSense D435 [51], enabling plant monitoring on both color and depth imaging modalities. These 2 imaging modalities are complementary in the study of plants, with visual, colorimetric, and spatial monitoring. The cameras are individually controlled by a Raspberry Pi 4 mini-computer [52], which triggers image acquisition at a given frequency via scheduled tasks.

This system has been presented in previous works [5–7], including in the context of plant abiotic stress studies [53]. The codes to pilot the acquisition is described and made available in [54]. In order to extend the scope of this system to biotic stress studies, the electronic components have been sealed, as illustrated in Fig. 1. This prevents contact between the electronic equipment and humidity, which can be very high in greenhouses. In plant pathology studies, the presence of pathogens in the ambient air means that all exposed equipment must be disinfected between tests. By grouping all these components together in a hermetically sealed case, disinfection of the case is possible, rather than the components themselves. Each case composed of 1 mini-computer and 1 camera costs around $500, all inclusive. The system, visible in Fig. 1, comprises 6 cameras, enabling plants to be monitored over a length of more than 3 m and a width of 1 m, with a high degree of overlap between the cameras’ field of view. In our context of high seedling density and batch-scale monitoring, each camera captures the evolution of about 18 plant batches in parallel, equivalent to 360 plants. As visible in Table 1, this throughput is huge compared with plant phenotyping systems in related works.

**Table 1.** Throughput and cost comparison with plant phenotyping models in related works based on grid of sensors

Source	Imaging modalities	Growth stage	Plants per camera	Cost per plant
[47]	RGB	Several leaves	1	∼$200
[86]	RGB-NIR	Several leaves	18	∼$11.1
[6]	RGB-Depth	First leaves	72	∼$6.9
[87]	RGB	Several leaves	∼60	∼$3.3
[48]	RGB	Several leaves	70	∼$2.9
Ours	RGB-Depth	First leaves	∼360	∼$1.4
[5]	RGB	First leaves	200	∼$1

### Datasets

Tests were carried out in greenhouses, based on the experimental protocol and imaging system introduced previously. Pepper plants, *Capsicum annuum*, were studied after inoculation with the TSWV pathogen [55,56]. Plants of the same varieties are grouped together in several plant batches, each composed of 20 plants. As illustrated in Fig. 1, the density of plants among the same variety repetition was high while distinct variety repetitions were spaced as far apart as possible to ensure minimum overlap with plants of different varieties. These test conditions enabled us to monitor plant growth of a specific variety repetition using a simple region of interest delimiting the corresponding batches. By default, rectangles were used to delimit the batches corresponding to variety repetitions. Sequences of *T* images of resolution (*W* × *H*) = (130 × 335) pixels were thus extracted for each batch of plants. A sample rate of 15 min was chosen to ensure high-frequency monitoring of plant growth. This corresponded to a periodicity *P* = 96 and a data flow of 96 images per day for each imaging modality monitored: color and depth. Environmental conditions, such as temperature and lighting, have repercussions on plant and pathogen development. The duration of the test was therefore subject to variability. On average, tests lasted 7 d, corresponding to sequences of *T* = 672 images.

For each batch of plants, a classification as resistant or susceptible, was established after visual inspection by 2 plant pathologists according to the experimental protocol defined in Experimental protocol. The resulting diagnoses served as ground truth for classification of resistant and susceptible varieties. The distribution of classes in the available annotated dataset is 208 batches of resistant plants and 50 batches of susceptible plants.

The growth of these varieties postinoculation was captured during the same tests for both classes, and therefore under identical conditions. Pathology tests, detailed in Table 2, were carried out on a wide range of pepper varieties, during different seasons of the year in different locations. These experimental constraints are representative of the wide variations in temperature and lighting that can occur in greenhouses.

**Table 2.** Description of the dataset composition for resistant and susceptible varieties

Pathosystem	Location	Sample rate (minutes)	Period	Batch distribution		Number of varieties	Plants per batch
				Resistant	Susceptible		
Pepper-TSWV	South of France	15	Jan. 2023	88	2	60	20
			Feb. 2023	45	0	36	
			Apr. 2023	30	36	53	
			May 2023	45	12	44	

**Fig. 2.**
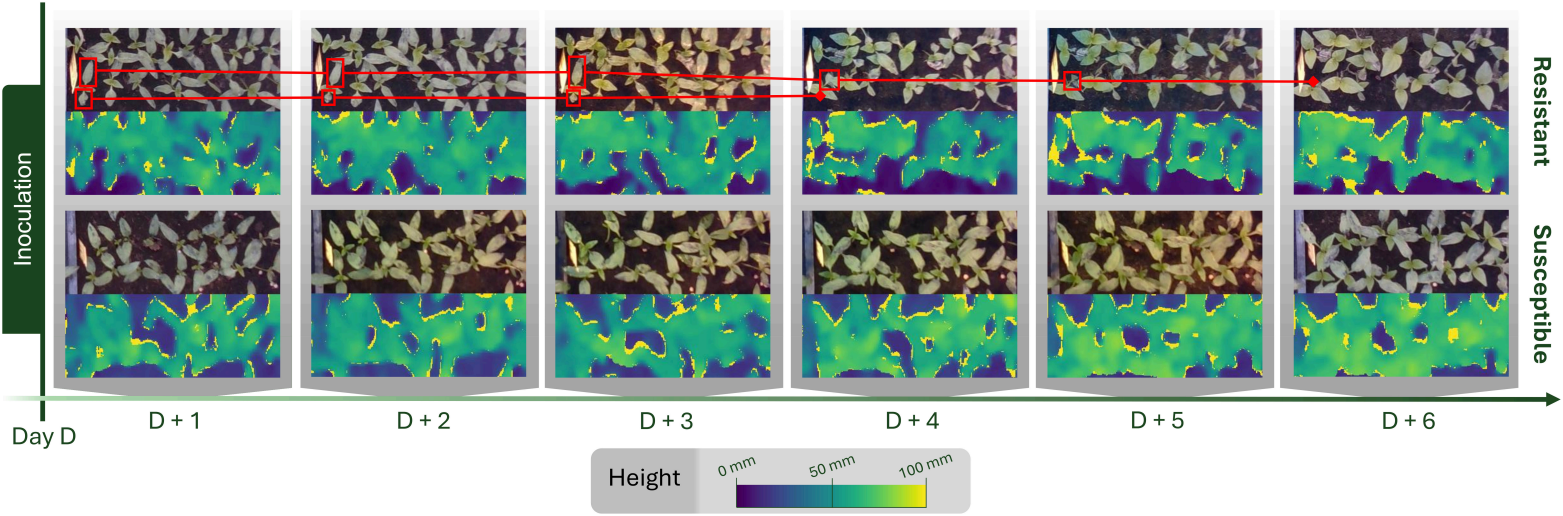
Daily time series postinoculation for 1 batch of resistant plants and 1 batch of susceptible plants. The cotyledon loss is visible on the development of plants from the resistant batch; an example of loss of 2 cotyledons from the same plant is represented and tracked by red boxes. See Movies S1 and S2 in the Supplementary Materials to get the full image sequence of each batch.

As visible in Fig. 2, the loss of cotyledon is perceptible in the red-green-blue (RGB) images. However, automating this drop detection based on RGB images would be a difficult task due to the strong occlusions and self-similarities between leaves which touch each others to constitute a canopy. We propose to investigate the value of the depth imaging modality for this detection of cotyledon loss. The depth imaging modality also has the advantage of providing continuous image sequences, unlike RGB sequences whose images cannot be acquired at night. In the remainder of this article, color images are then used to produce a visual ground truth to confirm that the expected changes visible on the depth images actually correspond to the observed loss of cotyledons.

### Qualitative signal analysis

The cameras produce raw images containing information of depth of the scene, seen from the sensor. Acquisitions over time $t\in \left\{0,\frac{1}{P},\ldots ,\frac{T}{P}\right\}$ in days produce depth time series noted as D. In order to base our frame of reference on the plants, each depth image $D\left(t\right)$ of the depth time series is converted into height image $H\left(t\right)$ in millimeters, as illustrated in Fig. 2. The top of the pots on which the plants are located is used as origin for height images. All values below this level are converted to zero. The binary mask *δ*(*t*) for canopy segmentation is obtained by considering that all pixels higher than zero belong to plants. We extracted 3 raw signals out of these time series of height images.

First, the raw signal of canopy surface S seen from top view is computed by counting the pixels corresponding to a plant according to the formula

$$S\left(t\right)=\sum_{\begin{array}{c} x\in ⟦1,W⟧\\ y\in ⟦1,H⟧ \end{array}}\delta \left(t\right)\left(x,y\right).$$ (1)

With the same approach, the raw signal of volume under canopy V can be calculated by the following formula

$$\begin{array}{c} \begin{array}{l} V\left(t\right)=\sum_{\begin{array}{c} x\in ⟦1,W⟧\\ y\in ⟦1,H⟧ \end{array}}H\left(t\right)\left(x,y\right)\cdot \delta \left(t\right)\left(x,y\right). \end{array} \end{array}$$ (2)

Then, height information contained by the 2D distribution of the height images is synthesized using the average height of the canopy. The raw signal of average height $\bar{H}$ is calculated according to the formula

$$\begin{array}{ll} \bar{H}\left(t\right) & =\frac{V\left(t\right)}{S\left(t\right)}. \end{array}$$ (3)

**Fig. 3.**
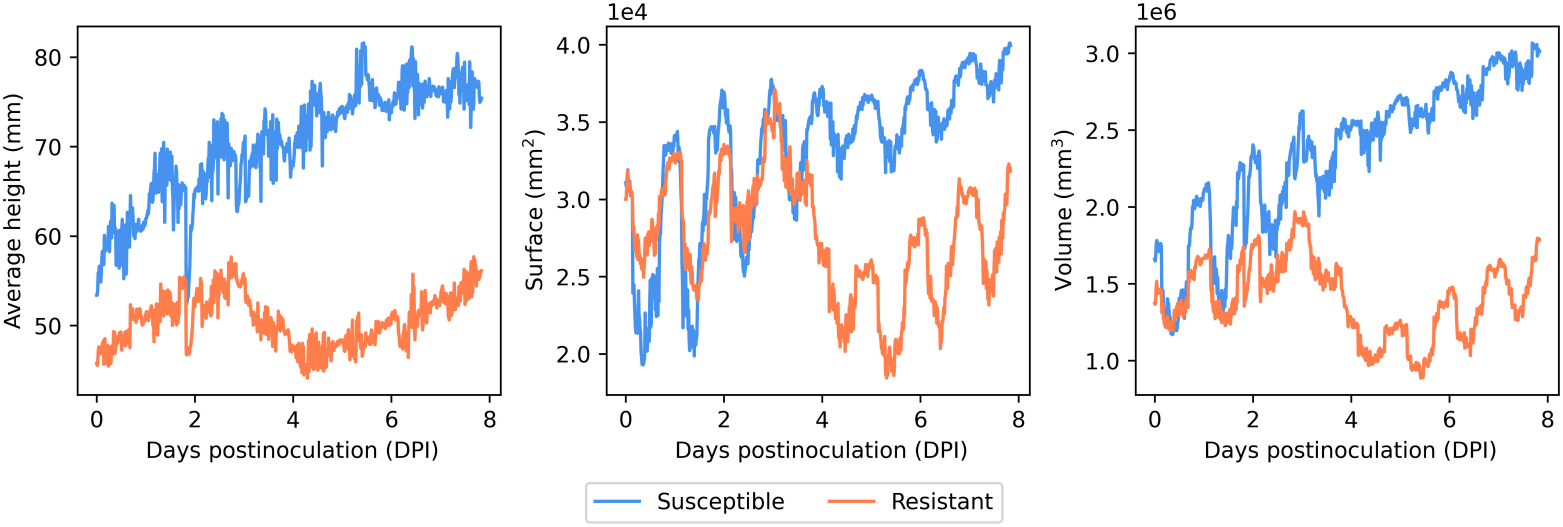
Raw signals of height $\bar{H}$, surface S and volume V from left to right, for an example of a resistant and a susceptible batch of plants.

Figure 3 illustrates a representative instance of such signals for an example of resistant and an example of susceptible batch of plants.

The raw signals of Fig. 3 can be decomposed into various components. The highest frequency corresponds to plant movements linked to growth of organs and appearance of new ones. We also observe some daily oscillations corresponding to the circadian cycles [57,58]. The lower-frequency movements correspond to the plant growth trend, which characterizes the long-term evolution of the plants. While in recent works, circadian cycles were shown of specific interest for the early detection of abiotic stress such as hydric or salt stress [8,53], they seem to be nondiscriminant for the biotic stress targeted here. A clear contrast appears when looking at the growth trend signal. Growth trend is monotonic for batches of susceptible plants. Conversely, we observe a nonmonotonic growth trend for batches of resistant plants. A spatial drop is observed before a recover of the growth.

**Fig. 4.**
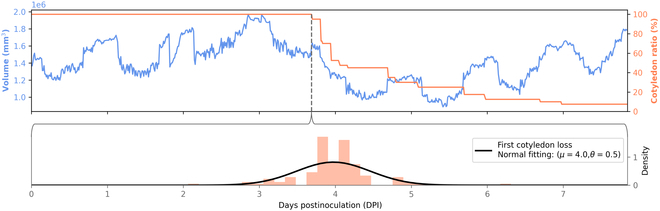
Analysis of timing correspondence between cotyledon loss and spatial drop. Above, correspondence between the observation of spatial drop on the raw signal of volume V and ground truth of cotyledon losses observed on the color image sequence for a resistant batch. Below, distribution of the temporal ground truth of the first cotyledon loss for the batches of resistant plants.

We investigated the cause of this drop. To this purpose, we have established the ground truth of the timing of cotyledon fall by visual detection using the RGB images. Figure 4 illustrates the temporal correspondence between the detected spatial drop and the first cotyledon loss appearing in the plant batch. This confirms that the detection of spatial drop effectively targets the cotyledon loss for the batches of resistant plants. The example of Fig. 4 suggests that the spatial drop appears a little upstream of the cotyledon loss, as we first detect the weakening of the cotyledons. Total necrosis or plant stalling occurs later. Finally, the volume starts to increase again around 5 d postinoculation, as the first plants to lose their cotyledons develop their first leaves more quickly.

### Design of a spatiotemporal feature space

In the previous section, we qualitatively analyzed the temporal patterns characteristic of batches of resistant plants by comparison with batches of susceptible plants. In this section, we construct a feature space suitable to quantitatively separate batches of susceptible and resistant plants.

Quantifying cotyledon loss can be negatively impacted by high-frequency patterns and circadian cycles [59] since they imply strong periodicity in the raw signals due to leaf oscillations. High amplitudes of spatial drop can be detected due to the amplitude of these circadian cycles without corresponding to the phenomenon of cotyledon loss. As this phenomenon lasts a few days, it corresponds to a lower-frequency signal. The spatial drop seems particularly noticeable at this frequency in Fig. 3. To extract growth trend information from the raw signals, we need a low-pass filter. A simple filter is the central moving average MA which leads to the removal of high-frequency patterns and periodic patterns like circadian cycles. A drop in the growth trend signal, stripped of high-frequency movements, is therefore highly representative of the cotyledon loss phenomenon.

For every raw signal *u* of periodicity *P*, the moving average MA*_P_*(*u*) is obtained according to the following formula

$$\begin{array}{l} MA_{P}\left(u\right)\left(t\right)=\frac{1}{P}\sum_{k=t-1/2}^{t+1/2}‍u\left(k\right). \end{array}$$ (4)

In our context, the periodicity *P* corresponds to the number of images acquired daily, since the rhythm of day/night cycles is synchronized to the periodicity. Growth trend signals are calculated for each raw signal introduced in Eqs. 1 to 3 and respectively noted $MA_{P}\left(S\right)$ for the growth trend signal of surface, $MA_{P}\left(V\right)$ for the growth trend signal of volume, and $MA_{P}\left(\bar{H}\right)$ for the growth trend signal of height.

The growth trend signals are used to quantify spatial drop and detect cotyledon loss. Drop detection in the growth trend signals consists in maximizing a drop amplitude. For each moment in the growth trend signal, we calculate the maximum possible drop amplitude from this point to the rest of the growth trend signal, excluding the past part. We then retain the characteristics that led to the steepest drop. Four features are calculated:

• *Onset O* corresponding to the starting time of the spatial drop period. It equates to the duration between pathogen inoculation and the beginning of spatial drop.

• *Duration D* corresponding to the time lag between the start and end of the detected drop period.

• *Absolute amplitude A_abs_* representing the spatial difference between the beginning and the end of the detected drop.

• *Relative amplitude A_rel_* representing the rate of amplitude of the spatial drop detected.

Extraction of such spatiotemporal features is summarized for every growth trend signal MA*_P_*(*u*) by the following formula

$$\begin{array}{c} \left\{\begin{array}{c} \left(O,D\right)=\text{argma}x_{\left(o,,,d\right)\in N^{2}}MA_{P}\left(u\right)\left(o\right)-MA_{P}\left(u\right)\left(o+d\right)\\ \;A_{\mathrm{abs}}=MA_{P}\left(u\right)\left(O\right)-MA_{P}\left(u\right)\left(O+D\right)\\ \;A_{\mathrm{rel}}=\frac{A_{\mathrm{abs}}}{MA_{P}\left(u\right)\left(O\right)}. \end{array}\right. \end{array}$$ (5)

**Fig. 5.**
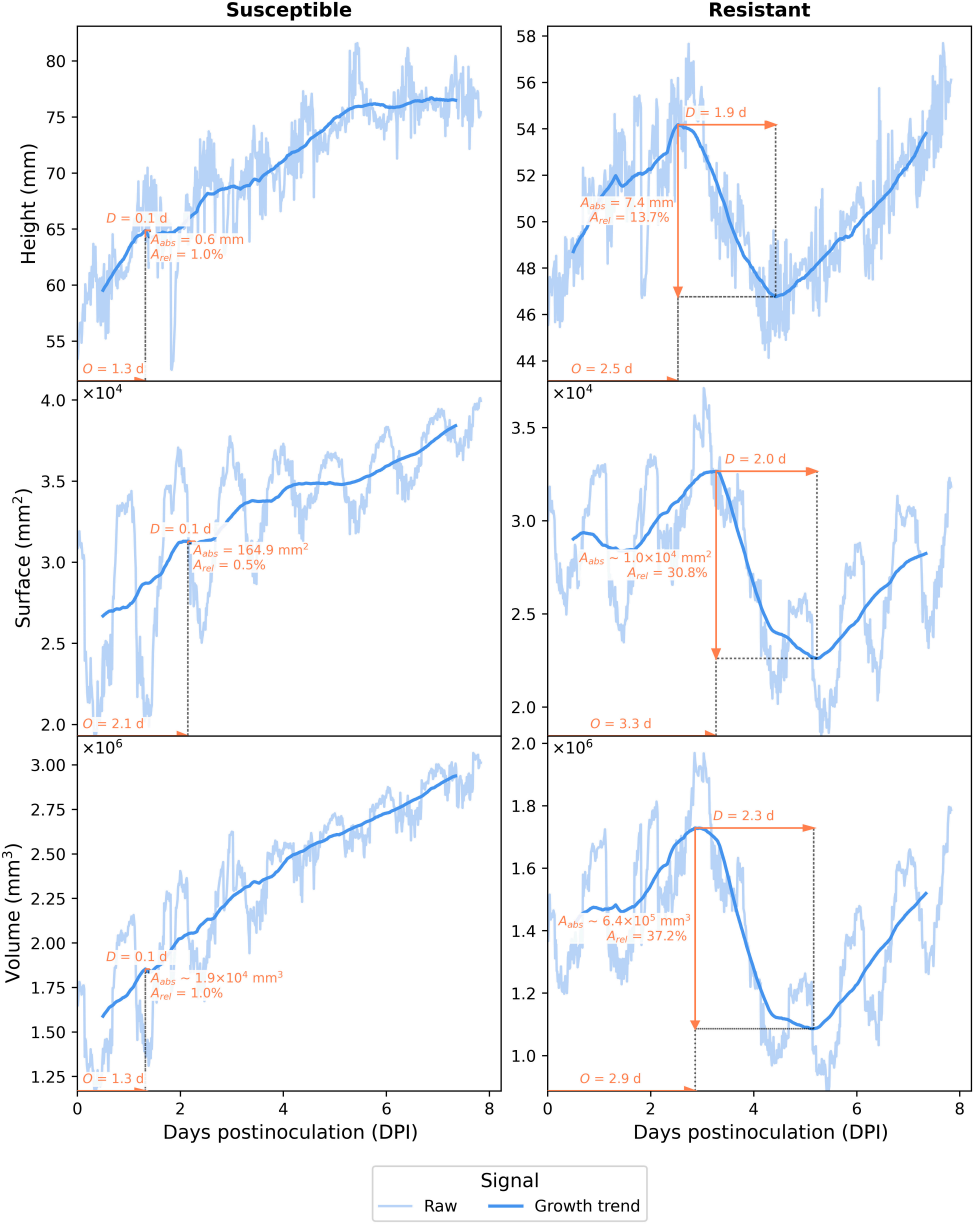
Example of feature extraction applied to 1 batch of susceptible plants (left column) and 1 batch of resistant plants (right column). Trend is obtained with moving average of daily period. *A_abs_*, *A_rel_*, *D*, and *O* correspond to the features defined in Eq. 5. From top to bottom, the raw and growth trend signals of height, surface, and volume.

Features are calculated for each growth trend signal $MA_{P}\left(\bar{H}\right)$, $MA_{P}\left(S\right)$, and $MA_{P}\left(V\right)$. An example of feature extraction is provided in Fig. 5.

**Fig. 6.**
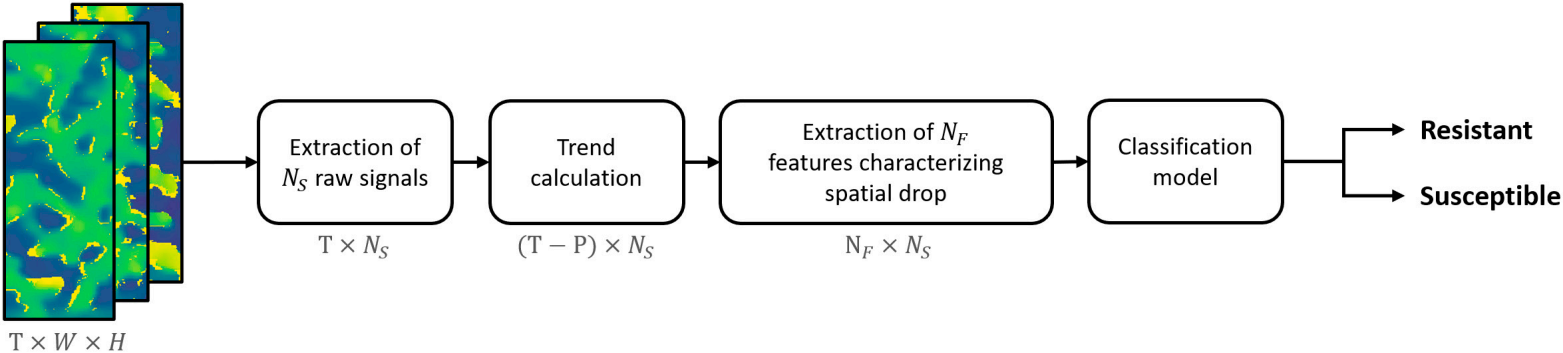
Process pipeline from depth image sequence to classification of batches of resistant and susceptible plants. Extraction of raw signals is done by Eqs. 1 to 3, trend calculation by Eq. 4 and extraction of features by Eq. 5. The size of the data is indicated for each step.

The dimension reduction corresponding to feature extraction can be quantified. Let *N_S_* = 3 be the number of growth trend signals and *N_F_* = 4 be the number of features. The feature space is therefore of dimension *N_F_* × *N_S_* = 12, which is very small compared to the dimension of the original image sequence space of dimension *T* × *W* × *H*. An overview of the pipeline with size of data at each step is represented in Fig. 6. On average, image sequences last 7 d and are made up of 672 images, all 130 x 335 pixels in size, taken at a frequency of 15 min.

The reduction of spatiotemporal information provided by the feature extraction with such parameters is a factor greater than 2.4 × 10^6^.

### Plant batch classification

Once the features have been extracted using the automated method presented in the previous section, a classification model can be elaborated for the separation of the resistant and susceptible distributions in the feature space. The overall pipeline is presented in Fig. 6.

Supervised machine learning models seem to be well suited to the amount of data involved. Three lightweight classification models have been tested:

• Random Forest [60,61] composed of 1,000 estimators with bootstrap method,

• Gradient Boosting [62–64] composed of 1,000 estimators,

• Support Vector Machine (SVM) [65,66] with Gaussian kernel.

To manage the small amount of data, 5-fold cross validation is used. To manage data imbalance, class weighting is done automatically according to the representativeness of plant batch classes in training for Random Forest and SVM models.

Three metrics are used to measure the quality of model predictions. Let TP, TN, FP, and FN be respectively the true positives, true negatives, false positives, and false negatives obtained by comparing the predictions with the ground truth. For classification task, the most conventional metric is the accuracy score [67] calculated using the formula

$$\begin{array}{l} \text{Accuracy}=\frac{\mathrm{TP}+\mathrm{TN}}{\mathrm{TP}+\mathrm{TN}+\mathrm{FP}+\mathrm{FN}}. \end{array}$$ (6)

In case of class imbalance, this metric is limited. Matthews correlation coefficient (MCC) [67], calculated with the formula

$$\begin{array}{l} \mathrm{MCC}=\frac{\mathrm{TP}\times \mathrm{TN}-\mathrm{FP}\times \mathrm{FN}}{\sqrt{\left(\mathrm{TP}+\mathrm{FP}\right)\left(\mathrm{TP}+\mathrm{FN}\right)\left(\mathrm{TN}+\mathrm{FP}\right)\left(\mathrm{TN}+\mathrm{FN}\right)}}, \end{array}$$ (7)

takes into account the representativeness of each class to provide a more robust measure in this context. Finally, a metric measuring the quality of prediction of each class is provided by the F_1_ score [67], calculated with the formula

$$\begin{array}{l} F_{1}=\frac{2\times \mathrm{TP}}{2\times \mathrm{TP}+\mathrm{FP}+\mathrm{FN}}. \end{array}$$ (8)

## Results

This section presents the results obtained by applying the methods presented in Design of a spatiotemporal feature space to the data presented in Datasets. First, the results relating to the extraction of spatiotemporal features are described. Next, the classification performance of the time series is presented, followed by the study of the robustness of the methods.

### Spatiotemporal feature extraction

**Fig. 7.**
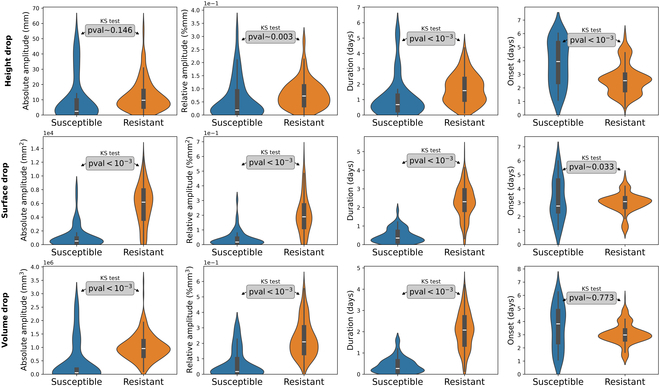
Distributions of features for batches of resistant and susceptible plants. Both density and boxplot are represented. From top to bottom, results obtained from growth trend signals of height $MA_{P}\left(\bar{H}\right)$, surface $MA_{P}\left(S\right)$, and volume $MA_{P}\left(V\right)$ are represented. Features, defined in Eq. 5, are represented in columns. The *P* values from the Kolmogorov–Smirnov (KS) test are indicated in the gray frames.

The method of feature extraction presented in Design of a spatiotemporal feature space has been applied to all batches of plants. An overview of the distributions in the feature space is represented in Fig. 7. Concerning features, absolute amplitude *A_abs_*, relative amplitude *A_rel_*, and drop duration *D* allow efficient separation of susceptible and resistant distributions. Onset *O* appears to be uniform for batches of susceptible plants, which confirms that the drop detected does not correspond to the loss of cotyledons. For the batches of resistant plants, the detected onset *O* is similar for each growth trend signal with an average of 3 d. This value is consistent with the spatial drop implied by the cotyledon loss. The distributions of features obtained for susceptible and resistant varieties reveal that some growth trend signals are more significant than others. The growth trend signal of height $MA_{P}\left(\bar{H}\right)$ does not seem to fully allow the distinction between batches of susceptible and resistant plants. However, the growth trend signal of surface $MA_{P}\left(S\right)$ and volume $MA_{P}\left(V\right)$ seem to reveal strong contrasts between the distributions of batches of susceptible and resistant plants. This result reflects the physiological reaction of resistance. The loss of cotyledons has little impact on the average height of the canopy, whereas surface is highly relevant, since cotyledons make up a significant proportion of the canopy surface at such growth stage, before they fall. As volume is linked to these 2 signals, it is also an accurate one.

To confirm the ability of the extracted features to effectively detect the cotyledon loss reaction at batch scale, a statistical test has been carried out. The Kolmogorov–Smirnov [68] test is performed to test the feature significance. The *P* values obtained for each feature are indicated directly in Fig. 7. This indicates that all features are significant at the 5% level except for the features of absolute amplitude with height signals and onset with volume signals. This test demonstrates the overall ability of the feature space to detect the cotyledon loss reaction at batch scale. As visible in Fig. 7, no single feature can completely separate the distributions because distributions of each feature remain overlapped. Simple thresholding on one of these variables cannot provide efficient batch classification. A more complex classifier, based on machine learning, must be used.

### Plant batch classification

Classification models presented in Plant batch classification has been applied to the feature space. The prediction quality of the models is evaluated according to the metrics defined in Eqs. 6 to 8. As represented in Table 3, the classification performance of the models is very high. The Random Forest model provides the best results, with the added advantage of easy interpretation of predictions. The corresponding accuracy score indicates 97% correct predictions. As the data are unbalanced, with around 4 times as many batches of resistant plants as susceptible ones, this metric is limited in this context. We then used the MCC, which confirms the good performance of the model, with a value of +91%. Finally, the F_1_ score was calculated to quantify the classification performance on each class. The results are good for each class, although the prediction of batches of resistant plants outperforms that of batches of susceptible plants. Class weighting, achievable for the Random Forest and the SVM models, improved stability of predictions, reducing the standard deviation of classification metrics.

**Table 3.** Metrics of classification model performance with 5-fold cross validation

Model	Class	F_1_ [0,1]	Accuracy [0,1]	MCC [−1,1]
Random Forest [60,61]	Resistant	0.98 ± 0.01	0.97 ± 0.02	0.91 ± 0.06
	Susceptible	0.93 ± 0.05		
Gradient Boosting [62–64]	Resistant	0.98 ± 0.01	0.96 ± 0.02	0.89 ± 0.03
	Susceptible	0.9 ± 0.02		
SVM [65,66]	Resistant	0.98 ± 0.01	0.96 ± 0.02	0.87 ± 0.06
	Susceptible	0.89 ± 0.06		

The use of a Random Forest model enables quantification of the frequency with which features are used for classification. This reflects the importance the model attaches to each feature for the task in hand. Table 4 indicates the importance of the features of Eq. 5 and the growth trend signals. Concerning growth trend signals, the growth trend of height $MA_{P}\left(\bar{H}\right)$ seems to be little used, unlike the growth trend of surface $MA_{P}\left(S\right)$ and volume $MA_{P}\left(V\right)$. Concerning the features, onset *O* is little used, whereas drop duration *D*, absolute amplitude *A_abs_*, and relative amplitude *A_rel_* are used extensively. This use of features corresponds well to those that mainly separate distributions in the feature space shown in Fig. 7.

**Table 4.** Importance rate of each feature (in rows) and growth trend signal (in columns) for the classification model. Bold formatting corresponds to high rates

%	Height	Surface	Volume	Total
Onset	2.2	2.6	3.3	8.1
Duration	2.8	**23.5**	**26.1**	**52.4**
Absolute amplitude	3.4	9.5	10.2	23.1
Relative amplitude	2.3	6.9	7.2	16.4
Total	10.8	**42.5**	**46.8**	

### Method robustness

**Fig. 8.**
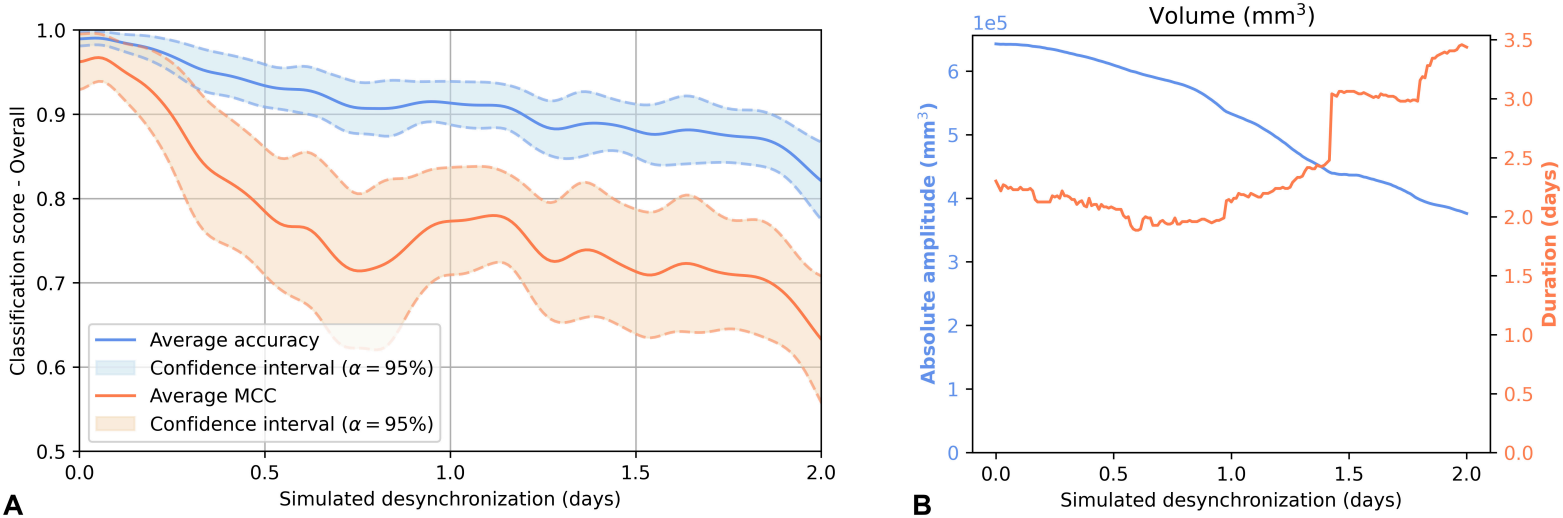
Impact of simulated desynchronizations of cotyledon loss on the feature space and the classification model. (A) Classification metrics with a model trained on raw data and inferred on desynchronized data. (B) Absolute amplitude and duration of the detected drop obtained by simulating desynchronizations on the growth trend signal of volume V for a batch of resistant plants.

The robustness of the methods presented in the previous section depends on the timing homogeneity of the hypersensitive reactions. Strictly equal growth and inoculation conditions should ensure minimum variance in the appearance of reactions in a given variety. It is this homogeneity that guarantees the effectiveness of batch-scale analysis. The more desynchronized the drop between plants, the more difficult it should be to detect on a batch scale. Desynchronization can occur mainly during inoculation, due to the time needed to inoculate all the plants in a batch. In order to test the robustness of our processes to such disturbances, we investigate adding simulated time shifts, supplementary to natural variability. Such simulations are done by concatenating each original depth image sequence with the same time-shifted sequence. Growth trend signal calculation and feature extraction is then applied to the raw signals obtained. As shown in Fig. 8B, these shifts logically reduce the amplitude of spatial drop because plants that have the earliest reaction then resume normal growth, while others continue to lose their cotyledons. On the other hand, at batch scale, this reduction in absolute amplitude *A_abs_* is accompanied by an increase in the duration *D* of the spatial drops, which is a high discriminating factor according to Fig. 7. Desynchronization creates modifications of the distributions in the feature space. This explains why the model trained on data without desynchronization performs well when inferring on slightly desynchronized data and poorly when inferring on highly desynchronized data, as visible in Fig. 8A. The drop in classification performance occurs after more than 2 h of simulated desynchronization. The time needed to inoculate all the 20 plants in a batch is about 1 min following the experimental protocol illustrated in Fig. 1. This is thus much smaller than 2 h. Consequently, one can reasonably conclude in the robustness of the method toward the practical sources of desynchronization of the plants within the batches in the process presented in this article.

**Fig. 9.**
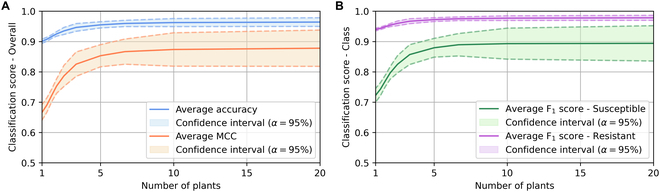
Classification metrics over number of plants in batches. (A) Overall classification metrics. (B) Classification metrics for each class.

Another matter to be addressed is the question of the influence of the number of plants in the batch on the performance of classification. The quantity of plants within batches is a parameter of the experimental protocol established by pathologists for their visual diagnosis. As the proposed method accesses growth kinetics, the quantity of information available is far greater than that of a pathologist establishing a diagnosis at a given moment. The required number of plants per batch can then be reconsidered. To assess the influence of this parameter, we simulated a variation of the number of plants per batch. A grid of the batches of 20 plants was extracted into boxes, and the classifier of the previous section, adapted to the box sizes, is applied to each box of the grid. Under the reasonable assumption that we have a uniform density of plants, the number of plants thus corresponds to the division of the original number of plants by the number of boxes in the grid. Figure 9 shows a monotonic evolution of the performance when the number of plants increases with an asymptotic plateau of performance for large enough number of plants. The quantity of 20 plants per batch defined in the protocol seems more than sufficient to characterize the spatial drop. Also, 10 plants per batch seem to maintain similar classification performance while doubling the throughput of batches analyzed by the system.

## Discussion

The results presented in the previous section demonstrate the effectiveness of the pipeline to get a high contrast between batches of susceptible plants and batches of resistant plants using depth imaging modality. In this section, we propose to discuss the advantages and limitations of this method.

To be relevant, automating diagnoses must demonstrate its added value. As we have seen, the performance of the classification model aim to match the quality of predictions made by plant pathology experts. On the other hand, a high added value is expected in terms of increasing the throughput of such diagnoses. To give a point of comparison, on average, pathologists need 1 min to diagnose 1 batch of 20 plants. The computation time of the proposed method has been investigated on the Raspberry Pi 4 mini-computer equipped with a quad-core CPU at 1.5 GHz and a standard storage device containing the image sequences. As visible in Table S1 in the Supplementary Materials, the computation time is around 2 s per plant batch from depth image sequences to classification. Deployed at the edge on a large scale, our method reduces diagnosis time by a factor of 30. It represents a real step forward in accelerating such plant pathology processes. Interestingly, less than 300 ms is required to obtain classification once the 1-dimensional signals have been extracted. Even lightweight classification models based on deep learning [69] require higher computation times on faster computers. Computation time of our method can be further reduced by advanced operating system configuration, multithreading and using a storage device offering much higher transfer speeds.

To be able to fully automate these tests in plant pathology, we need to provide a method that establishes a reliable diagnosis with as few errors as possible. Although the number of mispredicted batches is small, an investigation was carried out into the plant batches mispredicted by the classification model. As shown in Table 3, batches of resistant plants are better predicted overall than batches of susceptible plants. This cannot be explained by the imbalance between classes, since this imbalance is taken into account when the model is trained. We carried out a visual analysis on the RGB time series of susceptible batches predicted as resistant to identify the origin of these classification errors. These errors correspond to false positives in the detection of spatial drop. In practice, we observe that direct watering of plants strongly impacts plant vigor and induces artificial spatial drop. Generally speaking, it is reasonable to think that watering is a disturbance factor beyond classification errors. This explains why the drop amplitude distributions of susceptible and resistant batches are not strongly separated in the feature space and the low use of these features by the classification model. Consequently, the duration of this drop is an essential feature, since a drop linked to watering will be of very short duration compared with that linked to cotyledon loss. This source of disturbance can easily be corrected by watering the plants indirectly, automatically or using subirrigation. Removing watering-related errors leads to prediction accuracy over 99%.

The problem addressed in this article is a binary classification of susceptible and resistant batches. In specific cases, batches may be composed of both susceptible and resistant plants. Such batches are called segregating [70,71]. They appear in the early stages of plant breeding, mainly in prebreeding, when genotypes are not completely fixed. In this case, as the approach at batch scale does not allow direct quantification of the susceptible and resistant plants, it may seem limited. A study has been carried out on the ability of our method to diagnose segregating batches. Twenty-one segregating batches were acquired. This quantity is insufficient for training a 3-class model—susceptible, segregating, resistant—but it enables analysis of their projection in the feature space. As illustrated in Fig. S1 in the Supplementary Materials, an intermediate distribution between the susceptible and resistant distributions is obtained for spatial features. This result is explained by the lower number of plants affected by the cotyledon loss reaction in the segregating batches compared with the resistant batches. The temporal features logically seem to be identically distributed between the resistant and segregating batches. This study shows the potential of our method for detecting segregating batches while maintaining high throughput. Further work will be carried out by increasing the number of segregating batches and taking them into account in model training.

A study of the method’s generalizability to other pathosystems whose resistant plants show a hypersensitive reaction involving cotyledon loss has been carried out. Pepper plants inoculated with PMMoV [72] were monitored in tests involving differences in experimental protocol with batches of 30 plants with a lower density. Cotyledon loss in resistant plants appears slowed, as well as early leaf growth. The reaction therefore seems less marked, as illustrated in Fig. S2 in the Supplementary Materials. However, the pattern of spatial drop is preserved. Cotyledon loss is well detected, while the amplitude of spatial drop decreases and the duration of the phenomenon increases to around 8 d. The features extracted are still particularly suitable. This study validates that both the number of plants within batches and the transition from one pathosystem to another involve a transformation of the feature space that maintains its ability to efficiently separate the distributions of susceptible and resistant batches. The proposed method therefore seems robust to changes in pathosystem and experimental protocol.

The imaging system presented in Imaging system enables the acquisition of multiple imaging modalities: color, depth, and infrared. Since the aim of this article is to detect cotyledon loss on the scale of plant batches, the imaging modality of depth over time was chosen as it is particularly well suited to capture these spatial changes. To further extend the scope of this system, an interesting perspective is the combination of multiple modalities. Chlorophyll fluorescence was already studied with Pepper-TSWV [73] and Pepper-PMMoV [74], but this imaging modality is very expensive and not compatible with high throughput. Color and infrared modalities are affordable, compatible with such throughput, and renowned for their ability to capture strong contrasts with necroses [75–84], facilitating their detection. Such an approach would finally make it possible to automate the monitoring of an even wider range of hypersensitive reactions.

## Conclusion

Spatial monitoring of plants using a low-cost depth imaging system has been performed in plant pathology. A feature extraction targeting the hypersensitive reaction involving cotyledon loss in batches of resistant plants is carried out and the feature space constructed is highly discriminating for batches of susceptible and resistant plants. A classification model is trained, achieving 97% accuracy. This model is shown to be robust to usual desynchronization, number of plants in the batches and duration of the tests. These results are particularly impressive for a model based on a low-dimensional space of constructed features. This work provides further evidence that targeting known physiological phenomena can enable complex tasks to be carried out with lightweight methods that can be embedded on mini-computers.

## Data Availability

The data used in this study consist in RGB-Depth image sequences. They are available on request at cindy.torres@limagrain.com. The data are not publicly available due to privacy reasons.

## Supplementary Materials

Supplementary 1 Figs. S1 and S2 Movies S1 and S2 Table S1 Reference [85]
