# Supplementary material for: Affordable Phenotyping at the Edge for High-Throughput Detection of Hypersensitive Reaction Involving Cotyledon Loss
Source: Plant Phenomics. 2024 Jul 17;6:0204. doi: 10.34133/plantphenomics.0204 (PMC11251726; doi:10.34133/plantphenomics.0204)
Supplement: Supplementary 1 — Figs. S1 and S2 Movies S1 and S2 Table S1 Reference [85] [file plantphenomics.0204.f1.docx]

Movie S1. Example of RBG-Depth time series of a batches of resistant plants. The corresponding raw RBG-Depth hyperstack is provided as *resistant_sequence_raw.tif* in [87].

Movie S2. Example of RBG-Depth time series of a batches of susceptible plants. The corresponding raw RBG-Depth hyperstack is provided as *susceptible_sequence_raw.tif* in [87].

Table S3. Comparison of duration between manual and automated diagnosis. For automated diagnosis, the computation time is detailed step by step from depth image sequences to classification of plant batches following pipeline in Figure 6, running directly on a mini-computer [52] CPU core.

Fig. S4. Distributions of features quantifying spatial drop of surface including segregating batches.

Fig. S5. Extending the scope of the method of cotyledon loss detection to the Pepper-PMMoV pathosystem using spatio-temporal plant monitoring.
